# Driving Forces Sorted In Situ Size‐Increasing Strategy for Enhanced Tumor Imaging and Therapy

**DOI:** 10.1002/smsc.202100117

**Published:** 2022-02-04

**Authors:** Yue Wang, Wenyao Zhen, Xiue Jiang, Jinghong Li

**Affiliations:** ^1^ State Key Laboratory of Electroanalytical Chemistry Changchun Institute of Applied Chemistry Chinese Academy of Sciences Changchun Jilin 130022 China; ^2^ University of Science and Technology of China Hefei Anhui 230026 China; ^3^ Department of Chemistry Key Laboratory of Bioorganic Phosphorus Chemistry & Chemical Biology Tsinghua University Beijing 100084 China

**Keywords:** self-aggregation, self-assembly of small molecules, tumor imaging, tumor therapy

## Abstract

Nanoparticles (NPs) with diverse functionalities are widely used in tumor diagnostic and therapeutic. However, the therapeutic efficiency is unsatisfactory because of the limited penetration depth as well as short retention time in a solid tumor, resulting in the inaccessibility of NPs and low tumor treatment efficiency. Therefore, it is of extreme vital significance to design NPs for simultaneously realizing deep penetration and long retention. Considering that the smaller‐sized NPs may penetrate deeply in tumor and large‐sized NPs show enhanced retention, many kinds of in situ size‐increasing strategies of NPs have recently been developed for precise tumor imaging and therapy. In this review, the recent progress of stimuli‐induced size increasing of NPs in vivo according to the driving forces inducing the aggregation of the smaller entity into bigger one with ordered or unordered structures in disease sites is summarized. The biomedical applications of the size‐increasing strategy in the field of tumor imaging and therapeutics are introduced. Finally, the potential challenges underlying this strategy are briefly listed and its possible future clinical transformation directions are envisioned.

## Graphical abstract

In this review, the driving forces for in situ size increasing for enhancing tumors precise imaging and therapy are summarized and emphasized, which provide attractive points for clinical translation of precise diagnostics and effective therapeutics of diseases.

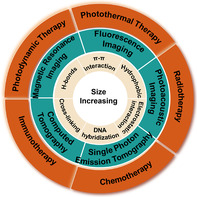

## 1 Introduction

Cancer, as a well‐known malignant disease in the world, remains the greatest challenge to humanity with the ever‐increasing mortality rate. The multifunctional nanoparticles (NPs) with high specific surface area, ease of modification, prolonged circulation time, and targeting properties have emerged for enhanced tumor theranostics.^[^ ^1^, ^2^, ^3^ ^]^ Early diagnosis, intraoperative localization, and postoperative imaging technologies that can be used to qualitatively or quantitatively characterize the progression of the disease at the cellular or molecular level are crucial for improving the curative effect of tumor therapy. Accordingly, several emerging imaging strategies based on NPs have been developed and exploited for clinical and/or preclinical purposes. For instance, as a distinctive diagnostic technology, photoacoustic imaging (PAI) that integrates the superiorities of the high sensitivity of fluorescence (FL) imaging^[^ ^4^, ^5^, ^6^, ^7^, ^8^, ^9^ ^]^ and relatively high penetration depth of ultrasonic (US) imaging improves the tissue penetration of the traditional FL depth, and can detect a depth of several centimeters.^[^ ^10^ ^]^ In addition, emission computed tomography (CT) (positron emission tomography [PET] and single‐photon emission CT (SPECT))^[^ ^11^, ^12^, ^13^ ^]^ can offer functional information on the diseased tissues with high sensitivity, greatly making up for the deficiency of traditional magnetic resonance imaging (MRI)^[^ ^14^, ^15^, ^16^ ^]^ and CT.^[^ ^17^, ^18^, ^19^ ^]^ The NP‐based contrast agents have significantly improved the imaging signals’ intensity of the existing imaging strategies to effectively visualize and distinguish diseases. In addition, constructing multifunctional NPs that can selectively deliver therapeutic reagents into the disease site while simultaneously generating unique imaging signals in vitro and in vivo is more conducive to the implementation of personalized diagnosis and treatment plans. Thus, many therapeutic strategies such as chemotherapy, radiotherapy (RT),^[^ ^20^, ^21^, ^22^ ^]^ photodynamic therapy (PDT),^[^ ^23^, ^24^, ^25^ ^]^ photothermal therapy (PTT),^[^ ^26^, ^27^, ^28^, ^29^ ^]^ and immunotherapy^[^ ^30^, ^31^, ^32^, ^33^ ^]^ have been developed and integrated into the diagnostic nanoplatform.

Although the development of the multifunctional theranostic nanoagents has made a great contribution to nanomedicine, the curative effect is unsatisfying. Indeed, only a small number of NPs can effectively penetrate and retard in the tumor to promote their function after systemic administration, while most NPs accumulate in the liver and spleen through nonspecific uptake, which greatly limits their development.^[^ ^34^, ^35^ ^]^ Therefore, designing NPs that can achieve high accumulation, deep penetration, and long retention appear to be particularly important for improving the therapeutic efficacy. As the basic feature of NPs, particle size is essential for regulating the entire delivery process in vivo.^[^ ^36^ ^]^ The NPs which are designed with diameters in the range of 20–200 nm may realize the prolonged blood circulation time and high tumor accumulation through the enhanced permeability and retention (EPR) effect.^[^ ^37^ ^]^ What's more, once entering into tumors, smaller‐sized NPs with the diameter smaller than 30 nm may penetrate deeply,^[^ ^38^ ^]^ while large NPs with the diameter around or above 150 nm are the desired choices trapped in the tumor to further facilitate their function.^[^ ^39^, ^40^, ^41^ ^]^ It seems intricate to design NPs with the constant size to achieve simultaneously penetration and retention in the tumor sites for enhancing the diagnostic and therapeutic effect. Recently, the in situ size‐increasing strategy has emerged as a solution to address these issues.

The in situ size‐increasing strategy is a process in which exogenous molecules or small‐sized NPs can penetrate deep into the tumor tissue and tend to grow into a relatively large size under the stimulation of pathological abnormalities in the tumor microenvironment (TME). According to the formation of the ordered or unordered structure, the strategy can be divided into self‐assembly and self‐aggregation processes. In the process, the smart exogenous molecules or small‐sized NPs consisting of responsive motifs can be cleaved by the abnormality of TME to alter their properties for further size increasing. Herein, the stimulations mainly include overexpressed enzymes, acidic microenvironment, and unbalanced oxidation−reduction state in TME. Upon the cleavage of a responsive group on initial molecules or relatively small‐sized NPs by the stimuli in the TME, the interactions such as hydrophobic, hydrogen bonding, π–π stacking, electrostatic interaction, DNA hybridization, or interparticle crosslinking reaction can drive them to interact with each other and grow into a relatively large‐sized structure. This strategy is expected to increase the concentration of diagnostic and therapeutic agents in the tumor site and prolong their exposure time, which can further contribute to highly specific and sensitive tumor imaging and therapy.

**Scheme 1:**
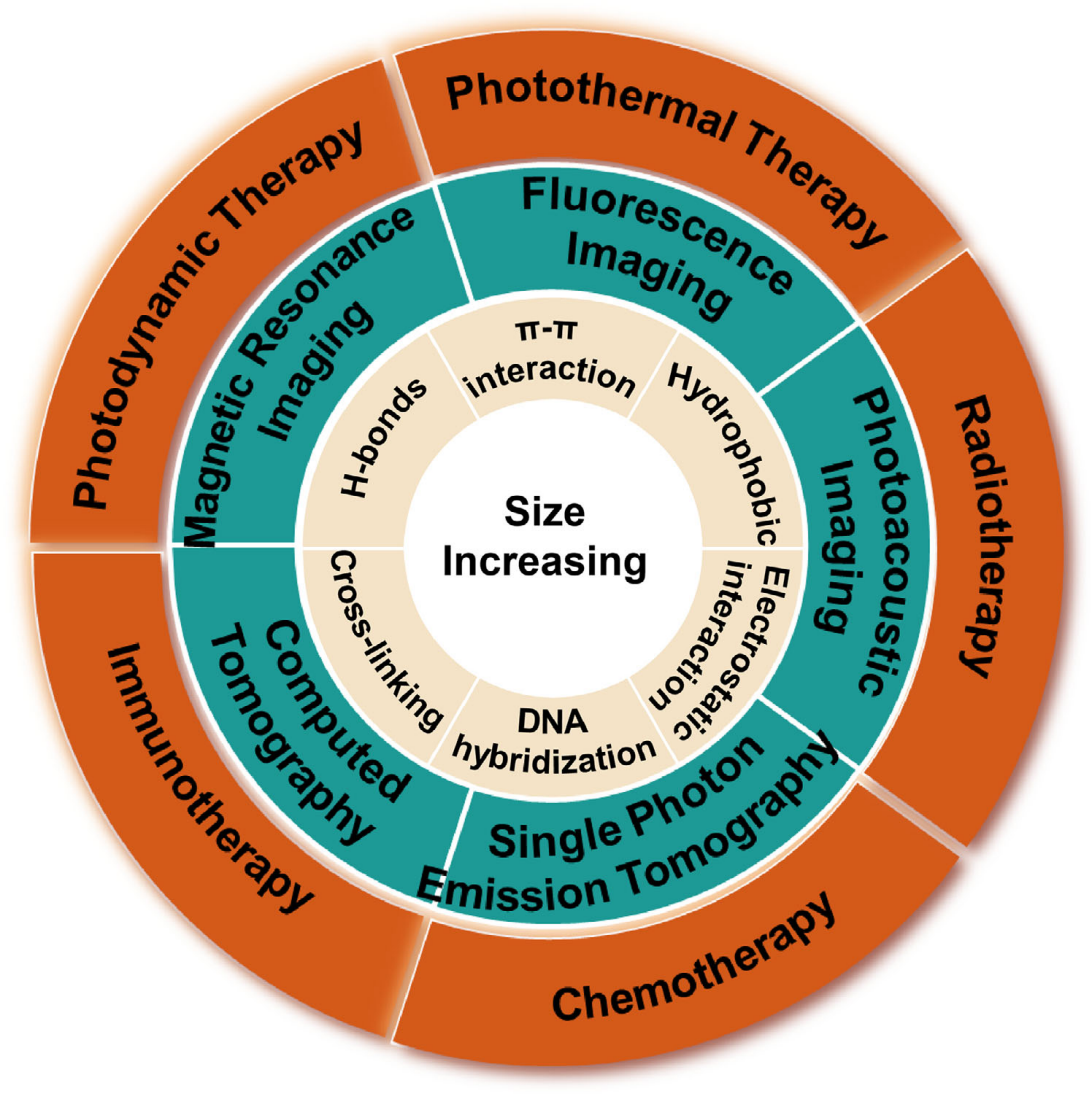
A schematic illustration of in situ size increasing of NPs driven by diverse interactions for enhanced tumor imaging and therapy.

The aforementioned molecular interactions are discussed as the driving forces for the in situ size increasing of NPs in this review, and the various stimuli such as enzyme, pH, and redox are also interspersed. Moreover, we also pay attention to their potential applications for enhancing tumor imaging and therapeutic effect (**Scheme** **1**, **Table** **1**). In the end, we briefly list the potential challenges underlying this strategy and envision the future possible clinical transformation directions of this strategy.

**Table 1:** A summary of in situ size‐increasing strategy and their biomedical applications

	Stimuli	Driving forces	Application	References
Self‐assembly	Matrix metalloproteinase‐2/9 (MMP‐2/9)	Hydrogen bonding, π–π stacking	FL imaging of RCC	[46]
	Caspase‐3/7	Hydrogen bonding	FL/PAI and chemotherapy	[47]
	Gelatinase	π–π stacking, hydrophobic interactions	PAI of tumor	[54]
	Alkaline phosphatase (ALP)	Hydrogen bonding, π–π stacking, hydrophobic interactions	FL imaging/PAI and PTT	[89]
	FAP‐α	Hydrogen bonding, π–π stacking	FL imaging of tumor	[90]
	MMP‐2	Hydrogen bonding, π–π stacking	MRI of tumor and PDT	[91]
	Cathepsin B	Hydrophobic interaction	FL/PET/CT imaging of tumor and chemotherapy	[92]
	pH	Hydrophobic interaction	FL imaging of tumor	[53]
	pH	Hydrophobic interaction	FL imaging and PDT therapy	[55]
	Glutathione (GSH) and furin	Cyanobenzothiazole‐cysteine (CBT‐Cys) click reaction, π–π stacking	MRI of tumor and chemotherapy	[93]
	pH	π–π stacking	FL imaging of tumor and immunotherapy	[59]
Self‐aggregation	pH	Electrostatic interaction	PAI of tumor and radiotherapy	[64]
	pH	Electrostatic interaction	Chemoradiotherapy	[65]
	Cathepsin B	CBT‐Cys click reaction	FL imaging and PDT therapy	[77]
	GSH	Maleimide‐thiol click reaction	MRI/SPECT imaging of tumor	[72]
	GSH and furin	CBT‐Cys click reaction	PTT therapy	[79]
	GSH and caspase 3/7	CBT‐Cys click reaction	MRI of tumor	[73]
	MMP‐2/9	[3 + 2] cycloaddition reaction	MRI of tumor	[94]
	MMP‐2	DNA hybridization	PET/PAI of tumor and chemophotothermal therapy	[69]

## 2 Self‐Assembly‐Based In Situ Size‐Increasing Strategy

The preparation of in situ size‐increasing NPs based on self‐assembly is emerging as one of the advanced strategies for enhancing tumor theranostics. In this process, the molecules or relatively small‐sized NPs consisting of functional and responsive motifs first penetrate deep into the tumor tissue, and then the interactions including hydrophobic, hydrogen bonding, and π–π stacking can drive their size transformation under the stimulation of pathological abnormalities in TME, ultimately growing into ordered large‐sized superstructures with stable and regular geometric appearance. Such an in situ size‐increasing strategy can achieve a higher signal‐to‐noise (S/N) ratio of contrast agents and prolong the exposure time of therapeutic agents, which is beneficial for effective tumor imaging and therapy.

### 2.1 Hydrogen Bonding‐Driven Self‐Assembly

Hydrogen bonding plays a crucial role in the formation of superstructures in the biological system. In addition, it also adds a powerful contribution to the construction of functional assemblies. Hydrogen bonding (X—H…Y) is formed by a hydrogen atom covalently bonded to an electronegative atom (X: e.g., F, O, N), being attracted by another adjacent negatively charged atom (Y: e.g., F, O, N).^[^ ^42^, ^43^ ^]^ It is extremely attractive due to their high fidelity and directionality. It is worth noting that a single hydrogen bond is weaker, while a structure with high stability and strong binding strength can be obtained via multiple hydrogen bonding arrays. For instance, natural proteins have specific secondary structures (e.g., α‐helix, β‐sheet), which are mainly driven by hydrogen bonding. In addition, β‐sheets are the most frequently used motifs which can drive the self‐assembly of peptides aggregated into fibrous structures among them.^[^ ^44^ ^]^

**Figure 1:**
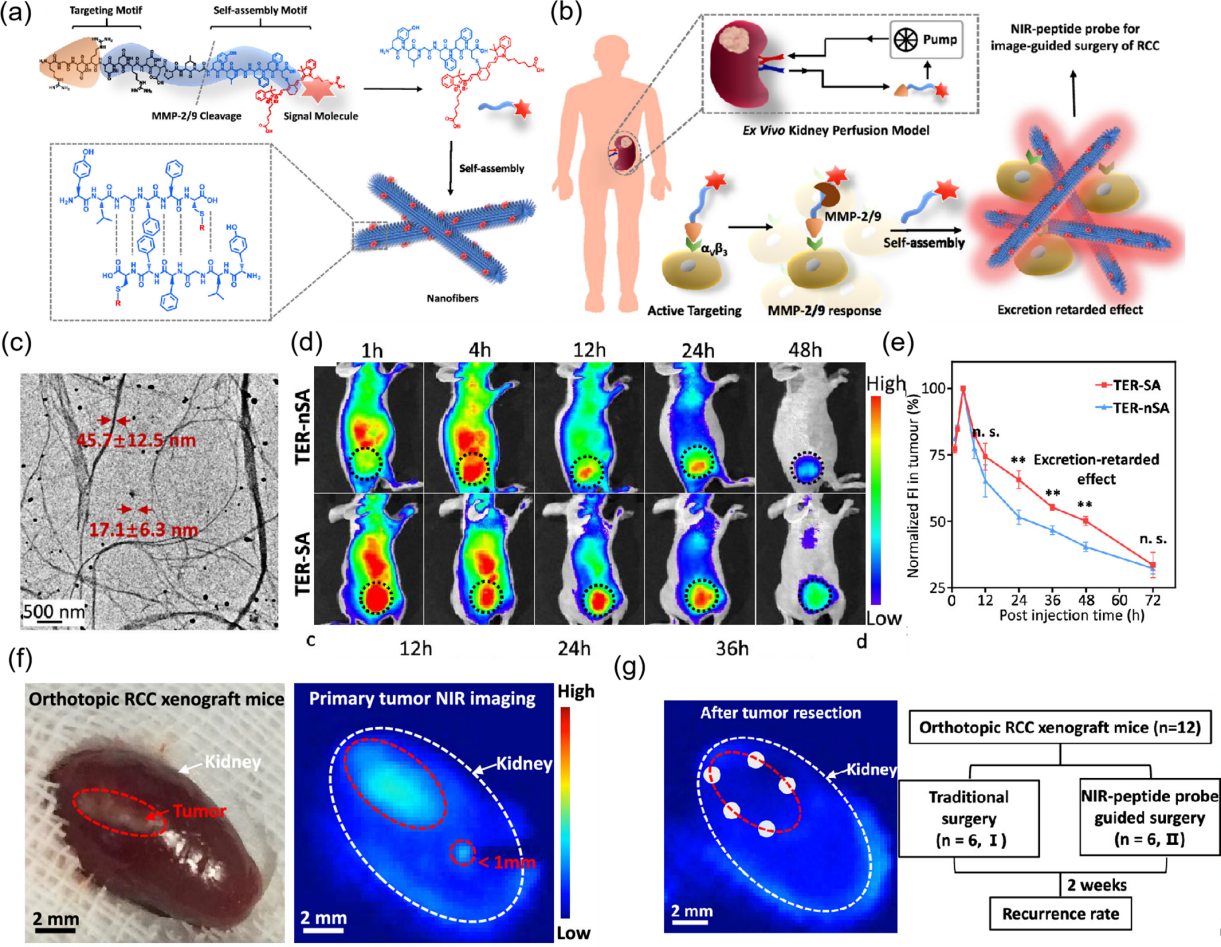
a) The structural composition of NIR peptide probe (RGDRDDRDDPLGYLGFFC (Cy), TER‐SA) and the mechanism of its in vivo self‐assembly. b) Mechanism of NIR peptide probe for high‐performance imaging‐guide surgery of tumor‐bearing kidney. c) TEM image of TER‐SA that self‐assembled into β‐sheet nanofibers at 24 h. d) NIR FL images of TER‐SA‐ and TER‐Nsa‐treated mice bearing 786‐O tumor at different time intervals and e) their normalized FL intensity. Data are presented as the mean ± standard deviation (SD) (*n* = 3). One‐way analysis of variance (ANOVA) was used for the indicated comparison: n.s. means no significance, **p < 0.01, ***p < 0.001. f) The macroscopic image of 786‐O tumor and its corresponding NIR FL images at 24 h postinjection. g) Random biopsy at the location of margin (*n* = 5) and the experimental design scheme. Scale bar = 2 mm. Reproduced with permission.^[^ ^46^ ^]^ Copyright 2020, American Chemical Society.

Along with tumor progression, some particular enzymes (e.g., matrix metalloproteinase, esterase, hyaluronidase, furin, caspase, and cathepsin) would be overexpressed in different tumor tissues and cells. Attributed to the highly specific substrate selectivity of the enzyme, a series of NPs with motifs that can be specifically recognized or cleaved by the overexpressed enzymes are constructed for further enzyme‐induced size increasing, which can simultaneously realize tumor‐specific targeting and enhance imaging intensity.^[^ ^45^ ^]^ FL‐mediated image‐guided surgery has the advantages of noninvasiveness, high specificity, and sensitivity. However, the nonspecific accumulation of the imaging agents in metabolic organs results in a high background signal, which greatly limits their clinical application. Xu and coworkers prepared a targeted enzyme‐responsive near‐infrared (NIR) peptide probe that could in situ self‐assemble into nanofibrous superstructures with β‐sheet domains via hydrogen bonding interactions, which have a tumor‐specific delay of excretion (TER) effect in tumor lesions and can be used to realize enhanced S/N ratio imaging for facilitating tumor resection of human renal cell carcinoma (RCC) (**Figure** **1**).^[^ ^46^ ^]^ The NIR peptide probe (RGDRDDRDDPLG**YLGFFC(Cy)**, TER‐**SA**) with modularized units includes 1) a tumor‐specific recognition motif (RGD); 2) an enzymatically cleavable linker (PLGYLG); 3) a self‐assembly motif (YLGFFC); and 4) a NIR signaling molecule (Cy) (Figure 1a). It can specifically recognize overexpressed α_v_β_3_ integrin in RCC and then is cleaved by the upregulated MMP‐2/9 in the TME. Thereafter, hydrogen bonding (N—H…O) directs the residue motifs (YLGFFC(Cy), SA) self‐assembled into nanofibers at the tumor site (Figure 1b). In vitro experiments exhibited that the SA can self‐assemble into nanofibers, and their diameters varied from 17.1 ± 6.3 to 45.7 ± 12.5 nm (Figure 1c). Further, nSA (YLGDDC(Cy)) peptide sequence which cannot self‐assemble into nanofibers was designed as the main component of the control group (RGDRDDRDDPLG**YLGDDC(Cy)**, TER‐**nSA)**. As shown in the in vivo experiment, TER‐SA and TER‐nSA were intravenously injected into tumor‐bearing mice; the FL signals of tumors treated by TER‐SA were continually enhanced within 12–48 h compared with control (Figure 1d,e). This strategy effectively increased the S/N ratio of tumor FL imaging, which can distinguish tiny lesions (<1 mm) (Figure 1f), and thus contributing to guide high‐performance tumor resection and finally reducing the recurrence rate of RCC surgery (Figure 1g). This tumor‐specific in situ self‐assembly strategy to form large‐sized superstructures demonstrates enormous potential for the clinical translational of image‐guided tumor surgery.

**Figure 2:**
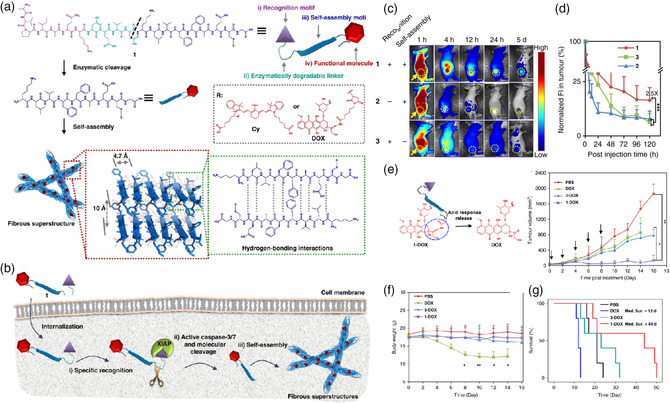
a) The structural composition of the TCASS system and its molecular design. b) The scheme of the in situ self‐assembly process of the TCASS system. c) Representative NIR FL images of mice bearing H460 tumor at different time intervals after intravenous injection (IV) injection of molecules 1, 2, and 3 and d) their normalized FL intensity. e) Schematic diagram of molecule 1‐DOX and its significant tumor growth inhibition. f) Body weight and g) survival curves of mice bearing H460 tumor after different treatments. Data are presented as mean ± SD (*n* = 5). One‐way ANOVA was used for the indicated comparison, *p < 0.05, **p < 0.01. Reproduced under the terms of the CC‐BY 4.0 license.^[^ ^47^ ^]^ Copyright 2019, The Authors, published by Springer Nature.

Besides using the overexpressed enzyme to trigger in situ size increasing for precise tumor imaging, it can also be conducted for both imaging and therapy through changing the functional motif. Zhao and coworkers developed a tumor‐selective cascade‐activatable self‐retained system (TCASS, AVPIAQKDEVDKLVFFAEC(Cy)G, molecular 1) for tumor imaging and therapy (**Figure** **2**).^[^ ^47^ ^]^ This system consists of an active‐targeting peptide sequence (AVPIAQK), a caspase‐3/7 cleavable linker (DEVD), a self‐assembly motif (KLVFFAECG), and a functional molecule (cyanine dye was conjugated for imaging or doxorubicin was conjugated for therapy) (Figure 2a). The AVPIAQK motif can specifically recognize the cancer‐upregulated X‐linked inhibitor of apoptosis protein (XIAP) and subsequently activated downstream caspase‐3/7. Then, hydrogen bonding directs the residue molecules cleaved by activated caspase‐3/7 that rapidly self‐assemble into nanofibrils with β‐sheet domains. After treatment with I‐labeled molecular 1 for 12 h, the formation and fibrous structures of nanofibrils inside H460 cells were observed by high‐resolution bio‐transmission electron microscope (bio‐TEM), and the diameter of the infinitely long nanofibrils was ≈12.6 ± 2.1 nm, which can be trapped in the tumor site (Figure 2b). Moreover, the strategy can also enhance the FL imaging of mice treated with molecular 1, and its FL signal was almost 2.5‐fold higher after administration for 5 days compared with control groups (Figure 2c,d). Due to the prolonged retention time in the tumor site, the Cy dye was replaced with a chemotherapeutic drug doxorubicin to achieve a higher antitumor effect (Figure 2e) and reduced systemic toxicity (Figure 2f), as well as the subsequent prolonged survival time (Figure 2g).

In addition, the KLVFF peptide sequence can also lead to the structure of NP change from spherical to nanofibers to facilitate their retention in the tumor site. Gao's group developed kinds of novel chimeric molecules consisting of a hydrophobic head (chlorin e6 (Ce6) or bilirubin (BR)), a self‐assembly peptide (FFVLK), and a hydrophilic tail (polyethylene glycol (PEG), which can form micelles in aqueous solution. When triggered by irradiation, the micelles can self‐assemble into nanofibers due to the hydrogen bonding of FFVLK, thus enhancing their retention to improve the chemophotodynamic therapeutic effect.^[^ ^48^ ^]^ Further, to amplify the PDT effect, Lu and Gao's group constructed a supramolecular nanodrug by the host−guest interaction between Ce6‐conjugated β‐cyclodextrin and ferrocene‐modified FFVLG_3_C−PEG conjugates. Upon being triggered by the endogenous reactive oxygen species (ROS) in tumor sites, the nanodrug can also self‐assemble into nanofibers to enhance the retention and generated more •OH and O_2_ to relieve hypoxia and amplify PDT efficiency.^[^ ^49^ ^]^ Notably, the nanofibers which were formed by self‐assembly through hydrogen bonding showed an obvious enhanced retention effect, sustained imaging capacity, and improved therapeutic efficacy.

### 2.2 Hydrophobic Interaction‐Driven Self‐Assembly

Hydrophobic interaction is the property of nonpolar molecules (or hydrophobic moieties of amphiphiles), which is also known as the hydrophobic effect and can drive these molecules to assemble into anhydrous domains in aqueous solutions.^[^ ^50^, ^51^ ^]^ It is also very important for the self‐assembly of synthetic amphiphilic block copolymers into nanostructures with various sizes and intelligent stimuli−response characteristics, which make them unique in drug‐delivery applications.^[^ ^52^ ^]^

**Figure 3:**
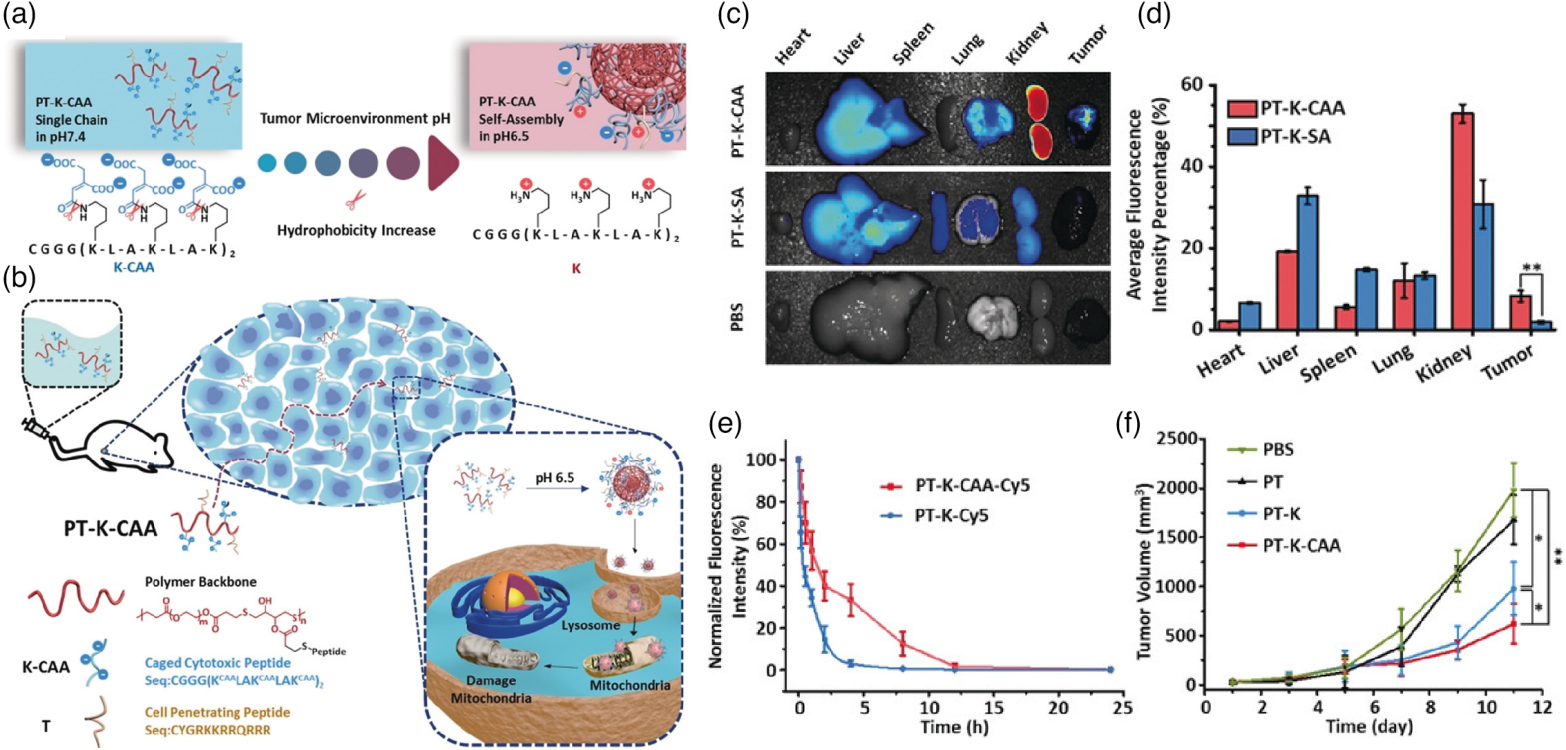
a) The hydrolysis process of CAA leads to an increase in the hydrophobicity of PT‐K‐CAA, which leads to its self‐assembly into nanoaggregates. b) Schematic diagram of penetration and self‐assembly of PT‐K‐CAA in acid TME for enhancing accumulation and improving therapeutic effect. c) NIR images and d) the quantitative analysis of FL intensity of organs at 24 h after different treatments (*N* = 3). e) The FL intensity of Cy5 in blood after treatment with Cy5‐labeled PPCs at different times. f) Tumor growth curves of different groups (*N* = 5). Statistical significance: *p < 0.05 and **p < 0.01. Reproduced with permission.^[^ ^53^ ^]^ Copyright 2019, Wiley‐VCH.

Relative to normal blood and tissues which show neutral pH (pH 7.2–7.4), tumor tissue exhibits a mildly acidic microenvironment with pH variation from 6.5 to 7.0 due to the excess production of lactic acid during the anaerobic glycolysis process. Moreover, the pH of lysosomes and endosomes is much lower ranging from 4.5 to 5.5. Based on this distinct acid microenvironment, plenty of acidity‐responsive NPs have been designed for tumor drug delivery. For instance, Wang's group designed polymer−peptide conjugates (PPCs) with properties of acidity‐triggered self‐assembly in TME for improving solid‐tumor penetration and antitumor efficacy activity (**Figure** **3**).^[^ ^53^ ^]^ Cis‐aconitic anhydride (CAA) which was regarded as a pH‐sensitive group was covalently linked to a cytotoxic peptide KLAK; then the products and a cell‐penetrating peptide TAT were conjugated onto poly (β‐thioester) backbones to form PT‐K‐CAA (PKC). The PKC was in a single‐chain state under normal physiological conditions, which can penetrate deeply into solid tumors. Once PKC reached the tumor, the acidity of TME would lead the hydrolysis of CAA and increase hydrophobicity of PKC, resulting in its self‐assembly into nanoaggregates via hydrophobic interactions at appropriate molecular concentrations (Figure 3a,b). In the self‐assembly process, the size of PKC grew from 18 to 90 nm in 12 h at pH 6.5. The NIR FL imaging experiment showed that the intensity of Cy5‐labeled PKC in tumor‐bearing mice was approximately fivefold enhancement compared with PT‐K‐SA with inability to form nanoaggregates at 24 h (Figure 3c,d). Moreover, owing to the negative charge of PKC, its half life can reach in about 2 h, thus prolonging its circulation time (Figure 3e). Further, the hydrolysis of CAA in the PKC can restore its a‐helix structure, allowing KLAK to exert its cytotoxicity toward mitochondria. Therefore, the in situ size‐increasing strategy significantly improved the therapeutic activity of PKC (Figure 3f). In addition to the pH‐induced self‐assembly for FL imaging, the enzyme‐induced self‐assembly through hydrophobic interaction, as its most important driving force for PAI, has already been conducted.^[^ ^54^ ^]^ In this work, Wang's group prepared a small‐molecule precursor (P18‐ PLGVRGRGD, P18‐1), including P18, gelatinase‐responsive peptide (PLGVRG), and targeting peptide (RGD). P18‐1 with RGD peptide can target overexpressed α_v_β_3_ integrins on cancer cell membranes, and subsequently the PLGVRG motif was cleaved by overexpressed gelatinase in TME, directing its self‐assembly into nanofibers in tumor sites for improving PAI signal intensity by sevenfold higher than the control molecule (P18‐2 with noncleavable linker) and subsequently enhanced therapeutic efficacy.

**Figure 4:**
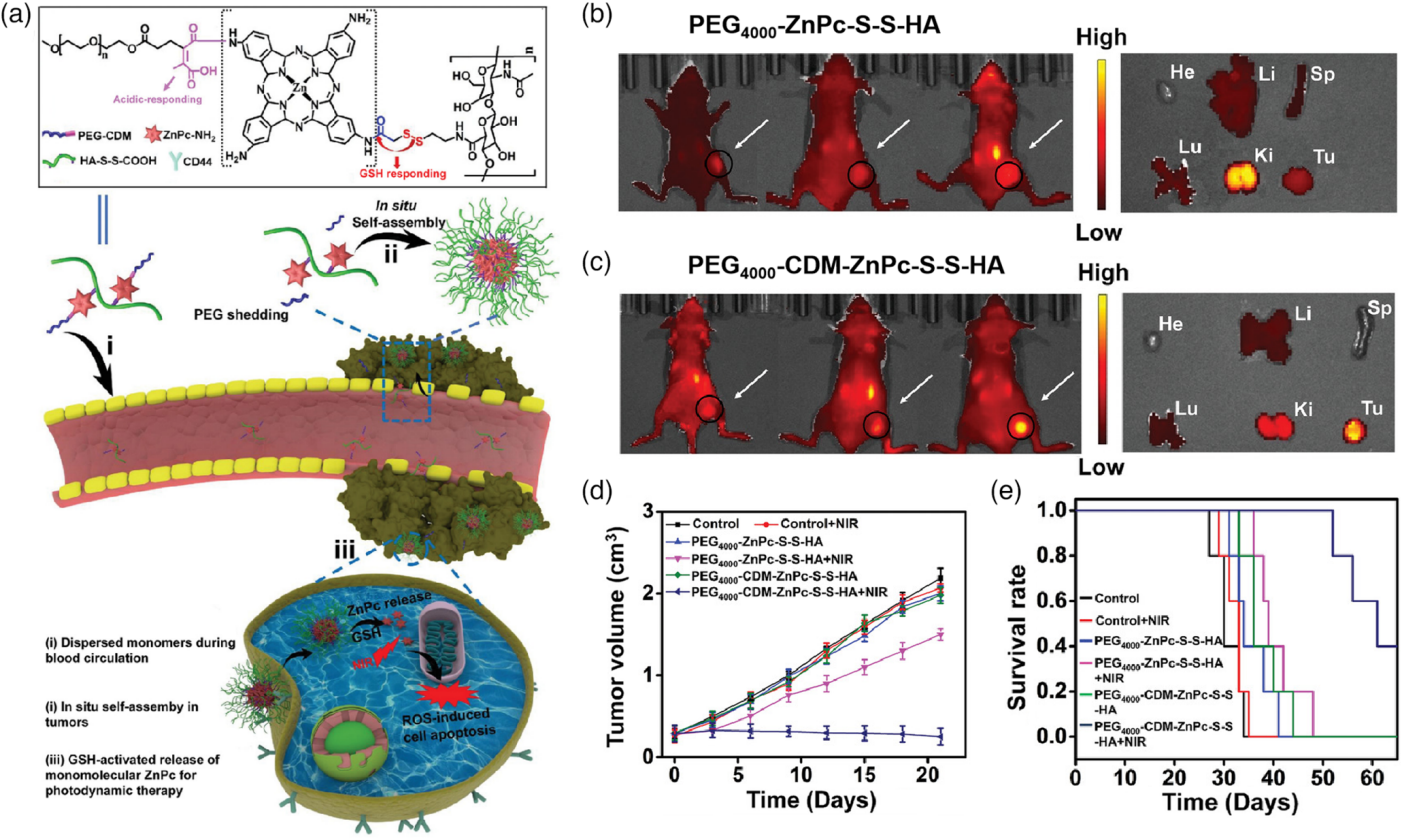
a) Chemical structure of PEG_4000_‐CDM‐ZnPc‐S‐S‐HA and the schematic illustration of in vivo self‐assembly strategy for enhanced PDT therapy. b,c) Whole‐body distribution of the different groups that were measured by NIR FL in 4T1‐tumor‐bearing mice models at different time intervals. d) The tumor growth curves and e) survival curves of different treatment groups. Reproduced with permission.^[^ ^55^ ^]^ Copyright 2020, Wiley‐VCH.

PDT, which utilizes PSs and light irradiation in the presence of molecular oxygen to produce ROS, is a promising modality for cancer treatment. Luo and Zhao's groups reported a novel acid‐triggerable in situ self‐assembly strategy with high tumor accumulation and retention for PDT (**Figure** **4**).^[^ ^55^ ^]^ Briefly, PS zinc phthalocyanine (ZnPc) and polyethylene glycol (PEG) were covalently linked via an acid‐cleavable maleic acid amide linker (CDM); then, the products conjugated to the hyaluronic acid (HA) chain through disulfide linkage to construct acid‐sensitive multisegment polymeric NPs (PEG_4000_‐CDM‐ZnPc‐S‐S‐HA). In the neutral phosphate‐buffered saline (PBS) environment, the NPs exhibited a monodispersed state (hydrodynamic size: 18 nm). The PEG segment with the ability of the enhanced blood circulation of the nanocarrier can be detached via the acid‐triggered breaking of the CDM linker when reaching acidic TME, and the rest of segments would undergo hydrophobicity/hydrophilicity balance changing and self‐assemble into a superstructure with a hydrodynamic size of around 200 nm (Figure 4a). Along this process, hydrophobic interactions acted as the main driving forces without additional external actuation. In vivo experiments exhibited that in situ formation of the large‐size (>200 nm) superstructure (ZnPc‐S‐S‐HA) enhanced Cy5 florescence signal intensity at the tumor region after mice were administered with PEG_4000_‐CDM‐ZnPc‐S‐S‐HA, compared with the nonacid‐responsive group (Figure 4b,c). The ZnPc‐S‐S‐HA superstructure was then internalized through HA‐facilitated endocytosis and further cleaved by the high GSH level to release of ZnPc in the tumor cells to facilitate PDT for amplifying the cytotoxic damage. Therefore, under NIR laser irradiation, the PEG_4000_‐CDM‐ZnPc‐S‐S‐HA inhibited the tumor growth effectively and prolonged survival time (Figure 4d,e). This acid‐triggered self‐assembly strategy can also be used for delivery of other hydrophobic agents and improve their antitumor efficacy.

### 2.3 Π–Π Stacking Interactions‐Driven Self‐Assembly

The π–π stacking interaction is also an important driving force for constructing diversified complexes, which are widely present in biological systems. It is a kind of weak interaction that often occurs between two relatively electron‐rich and electron‐deficient aromatic rings, which exhibit a special spatial arrangement. Up to now, peptide derivatives with aromatic groups, such as FF, P18, bis‐pyrene (BP), naphthalene, and carbobenzyloxy,^[^ ^56^, ^57^ ^]^ have been used to establish self‐assembled nanomaterials via π–π stacking for tumor therapy.

**Figure 5:**
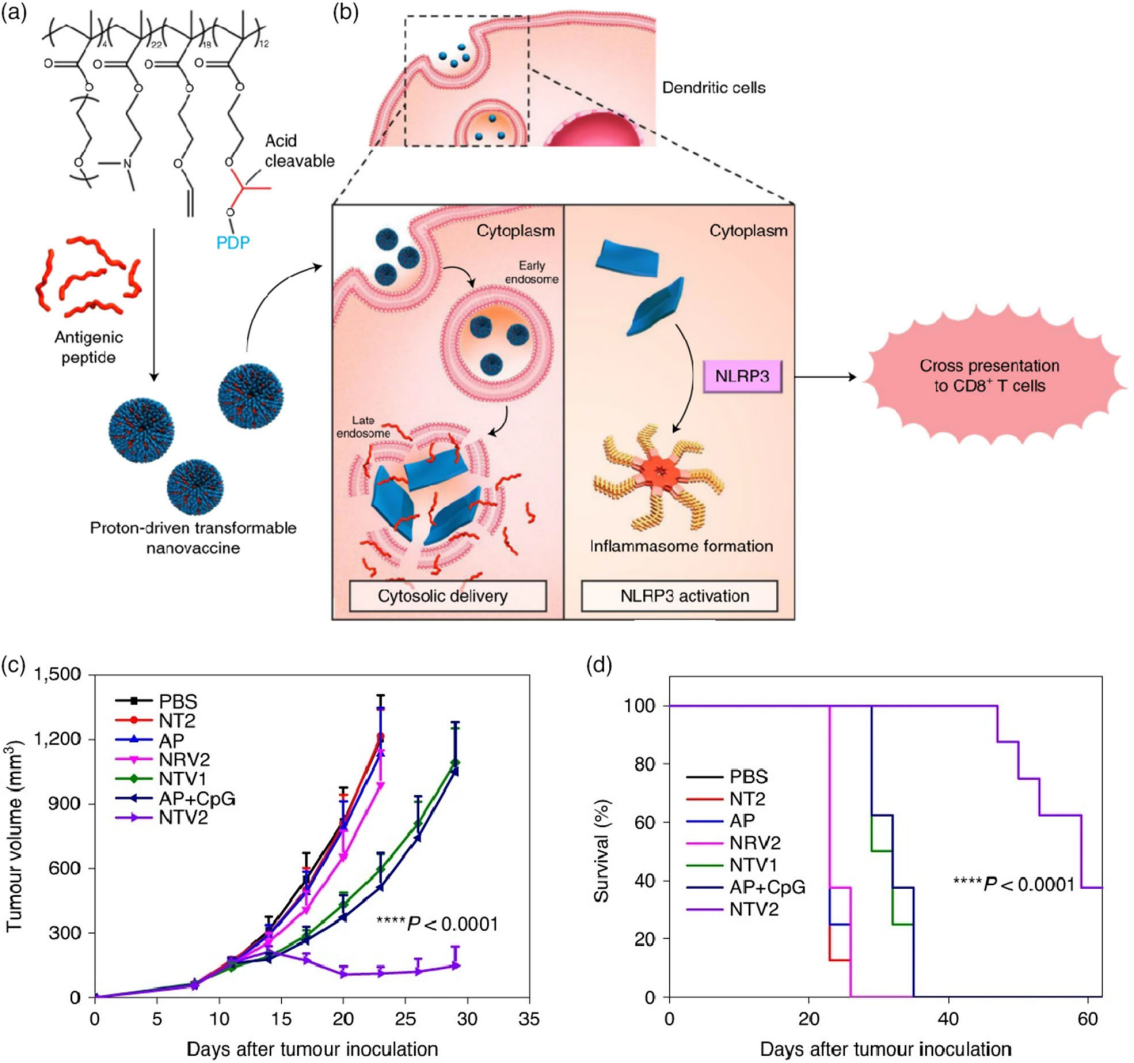
a) The nanotransformer‐based vaccine (NTV) consists of a pH‐sensitive polymer–peptide conjugate‐based nanotransformer (NT) and AP. b) Once the NTV entered into the acidic endosomal environment (pH 5.6) in DCs, the PDP will be fast cleaved, and the released PDP will reassemble into nanosheets with the size of 5–8 μm. The large‐sized nanosheets lead to mechanically disrupting the endosomal membrane and deliver the AP directly to the cytoplasm to realize effectively antitumor immunity. c) The antitumor effect and d) survival curves of the B16F10‐OVA tumor model. Reproduced with permission.^[^ ^59^ ^]^ Copyright 2020, Springer Nature.

Tumor immunotherapy that depends on the mobilization of the immune system to kill tumor cells has become a hopeful cancer treatment. The nanovaccine shows great potential in improving the efficacy of immunotherapy due to its unique advantages of codelivery of antigens and adjuvants and has become an essential form of immunotherapy.^[^ ^58^ ^]^ To stimulate the strong immune response, effective cytoplasmic delivery of tumor antigens is particularly important, but it is usually limited by endosome capture. Liang and Li’ groups developed a novel nanotransformer‐based vaccine (NTV), which could reassemble into large‐sized superstructures under the acidic TME (**Figure** **5**a). Then, the large‐sized superstructure can further mechanically destroy the endosomal membrane, and antigenic peptide (AP) was effectively delivered into the cytoplasm for tumor immunotherapy.^[^ ^59^ ^]^ Different amounts of hydroxylated naphthalene‐conjugated peptide (NDP–OH) or hydroxylated pyrene‐conjugated peptide (PDP−OH) were conjugated to the hydroxyl groups of p(DMAEMA_22_‐OGEMA_4_)‐*b*‐p(MAVE)_30_ via a pH‐responsive acetal bond, and an AP was loaded to form NTVs (NTV1 or NTV2). The NTV was spherical and had a diameter of ≈100 nm at pH 7.4. However, once NTVs entered acidic endosomes of dendritic cells (DCs), the NDP and PDP peptides were released due to the cleavage of the acetal bond. Then, the released peptides have large aromatic structures, which can induce π–π stacking among molecules to further reassemble into nanofibers (NDP) or nanosheets (PDP) with the size of several micrometers, respectively (Figure 5b). Thus, in the intracellular distribution experiment, the FL signal intensity of AP in cytosolic enhanced remarkably in NTV‐treated DCs, compared with free AP and pH‐unresponsive NPs (NRV1 and NRV2). This indicated that acidic‐responsive NTVs successfully mechanically disrupt the endosomal membrane and deliver AP directly to the cytoplasm, which will promote DC maturation and enhance crosspresentation of AP to CD8^+^ T cells by activating the specific inflammasome pathway and ultimately realizing efficient antitumor immunity. Owing to the strong immune response and sustained antigen crosspresentation induced by NTV2 (self‐assembly into nanosheet), it effectively inhibited tumor growth (Figure 5c) and significantly prolonged survival time in the B16F10‐OVA model (Figure 5d).

## 3 Self‐Aggregation‐Based In Situ Size‐Increasing Strategy

In addition to self‐assembly into order structure, some molecules or small‐sized NPs could undergo transformation through electrostatic interaction, DNA hybridization, or interparticle crosslinking reaction under TME stimuli to further form a large and unordered structure, named as self‐aggregation‐based in situ size‐increasing strategy.

### 3.1 Electrostatic Interaction‐Driven Self‐Aggregation

Electrostatic interaction is the driven force between any two appositely charged molecules, which has been used for the in vitro construction of NPs for delivery of chemotherapeutics,^[^ ^60^, ^61^ ^]^ gene,^[^ ^62^ ^]^ and antigenic peptide^[^ ^63^ ^]^ to effectively inhibit tumor growth. Moreover, it can also drive the self‐aggregation of NPs under acid stimuli for enhanced tumor imaging and therapy. Upon the electrostatic interaction of NPs with opposite surface charges, they will further aggregate in the tumor site because of increased uptake driven by sedimentation and decreased extracellular efflux.

**Figure 6:**
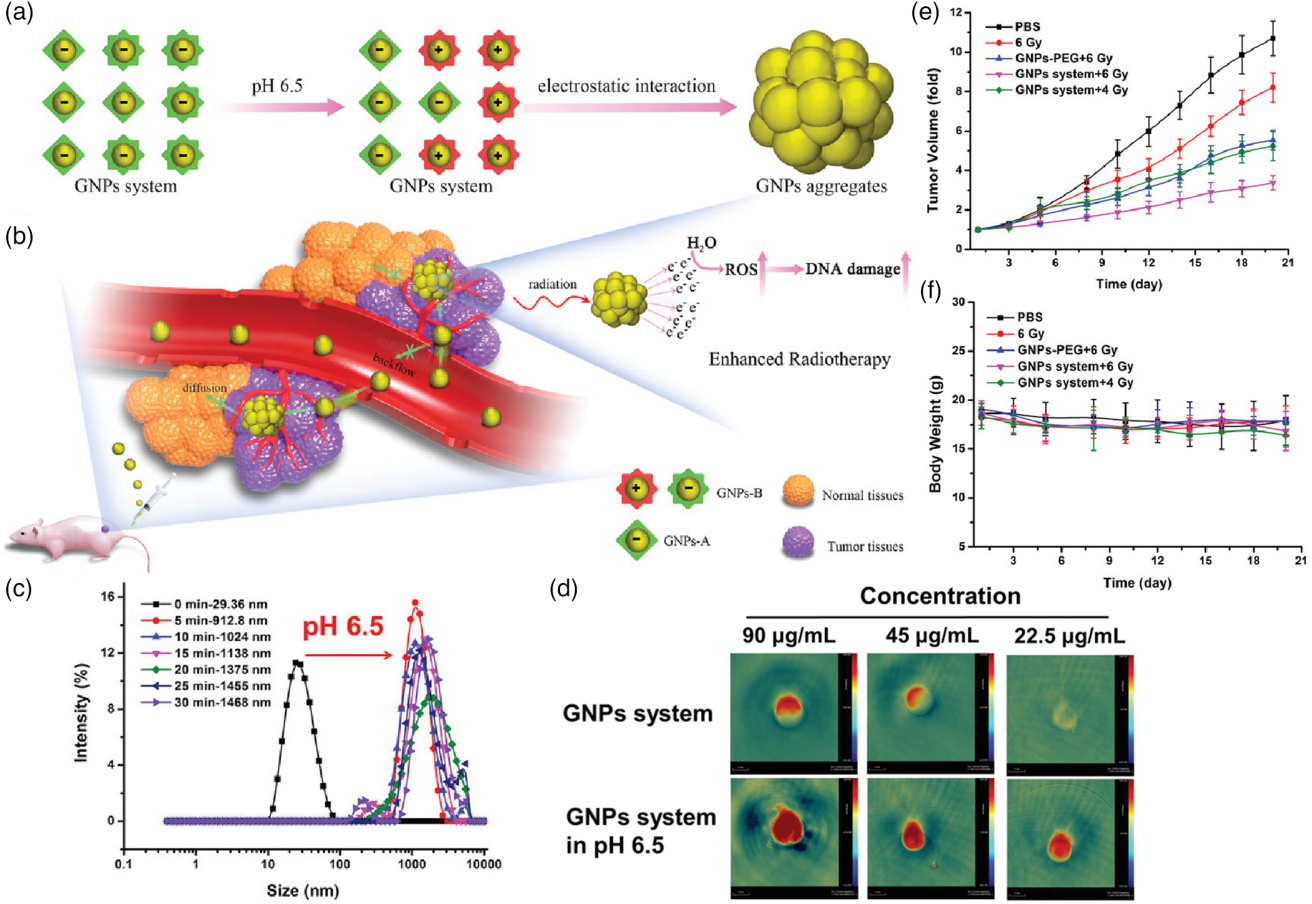
a) Scheme illustration of the acid‐induced self‐aggregation of GNPs. When the GNPs enter into the acidic environment, GNPs‐B undergo a charge reverse, where electrostatic interaction drives it to interact with GNPs‐A to form nanoaggregates. b) After intravenous injection of GNPs system, the in situ self‐aggregation occurs for improved tumor retention and enhanced RT efficiency. c) The size increase of GNPs at pH 6.5 verified by DLS. d) The PAI of GNP system at different concentrations, pH 7.4 and 6.5, respectively. e) Tumor volume and f) body weight curves of the mice treated with different groups. Reproduced under the terms of the CC‐BY 4.0 license.^[^ ^64^ ^]^ Copyright 2019, The Authors, published by Wiley‐VCH.

Recently, Liu's group reported acid‐triggered in situ self‐aggregation by developing two types of small‐sized gold NPs (GNPs) that modified with peptide to form GNPs‐A and GNPs‐B, respectively (**Figure** **6**a,b).^[^ ^64^ ^]^ Under extracellular acidic conditions (pH 6.5), the negatively charged groups on the surface of GNPs‐B could be detached and its surface charge reversed into positive, and then electrostatic interactions driving it interact with GNPs‐A with negative surface charge; finally, the nanoaggregates were formed with the sizes of 1468 nm (Figure 6c). Moreover, GNPs (AuNPs) are considered as the most attractive contrast agents for PAI, due to their unique surface plasmon resonance characteristics and high extinction coefficients. As shown in Figure 6d, the PA signals of the GNP system (90, 45, 22.5 μg mL^−1^) at pH 6.5 in vitro were improved remarkably with increased concentration compared with that of pH 7.4. What is more, attributed to the high X‐ray absorption of AuNPs, the GNP aggregation would be regarded as an excellent radiosensitizer for enhancing RT efficiency. The antitumor experiment indeed demonstrated that in situ self‐aggregation of the GNP strategy could reduce radiation dose, while enhancing radiosensitization, which effectively inhibited the tumor growth with no significant change in body weight (Figure 6e,f).

However, the efficiency of this mixed‐mode‐responsive self‐aggregation strategy is strictly limited by the concentration of two kinds of NPs that reach the tumor site. Recently, Yuan's group reported smart dual pH‐responsive NPs (Au@PAH‐Pt/DMMA), which can self‐aggregate for the combined chemoradiotherapy.^[^ ^65^ ^]^ In the normal physiological environment, Au@PAH‐Pt/DMMA was hydrophilic. When it is exposed to acidic TME, the acid‐labile molecules DMMA on its surface will be removed.^[^ ^66^, ^67^ ^]^ Subsequently, the destruction of DMMA leads to the exposure of the protonated amine group with positive charge, and the electrostatic interaction between NPs with opposite charges would drive their self‐aggregation into a large‐sized structure of 3 μm. When exposed to a low dose of X‐ray, these aggregates could still exhibit a high tumor inhibition rate with decreased drug administration frequency. Taken together, this self‐aggregation strategy driven by the electrostatic interactions could effectively improve the efficiency of tumor treatment tumor and provide a potential method for their clinical translation.

### 3.2 DNA Hybridization‐Driven Self‐Aggregation

**Figure 7:**
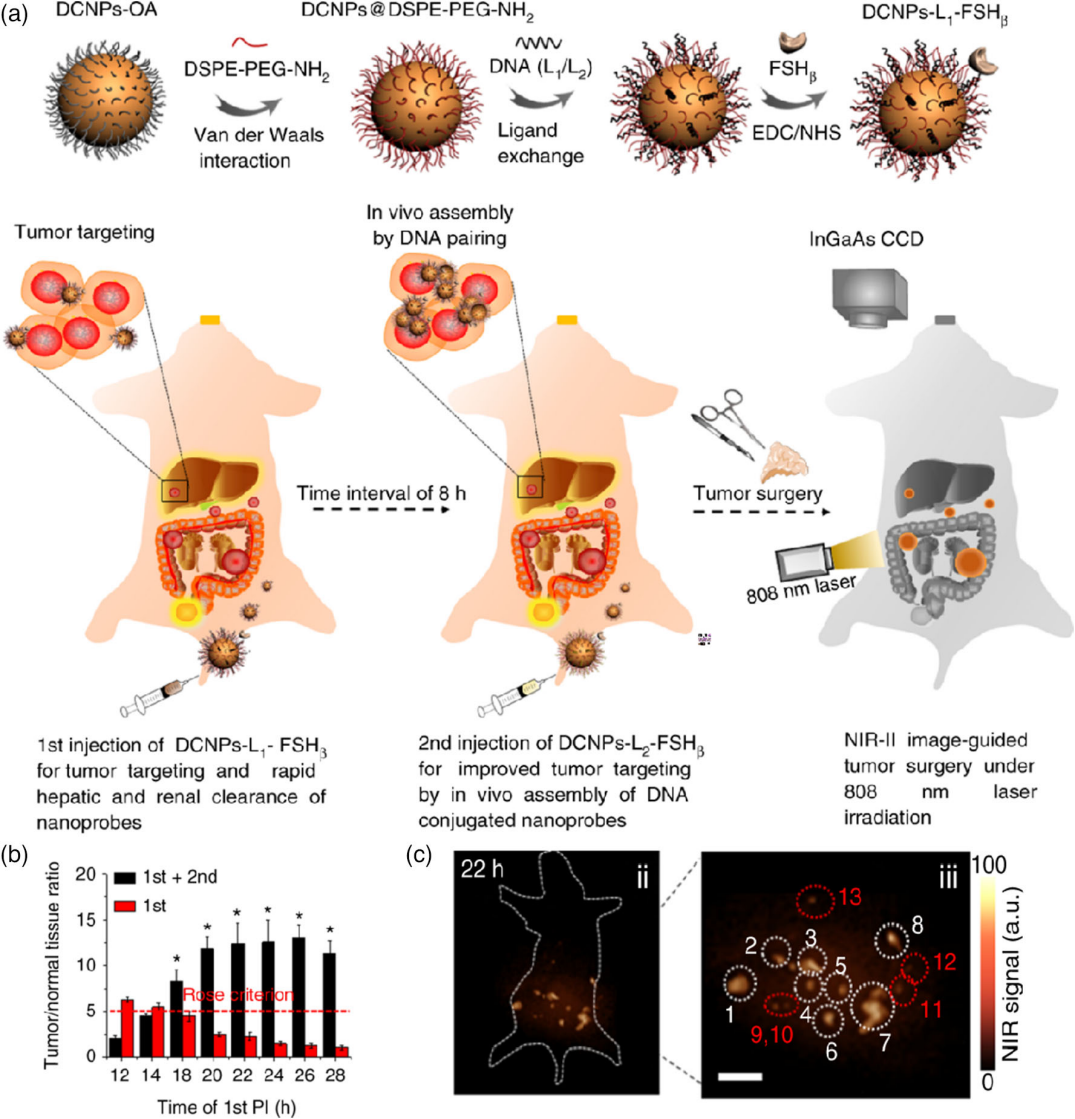
a) Schematic illustration of NIR‐II nanoprobes’ (complementary DNA and FSH_β_‐modified DCNPs) construction for NIR‐II bioimaging guiding ovarian metastasis surgery. Following the two‐stage injection, the in vivo‐formed aggregates significantly enhanced NIR‐II image‐guided surgery. b) T/N ratio of single injection and two‐staged sequential injections, respectively. c) Corresponding NIR‐II FL bioimaging of human ovarian adenocarcinoma peritoneal metastases model. Scale bar, 1 cm. Reproduced under the terms of the CC‐BY 4.0 license.^[^ ^88^ ^]^ Copyright 2018, The Authors, published by Springer Nature.

DNA hybridization refers to DNA molecules with unique complementary base sequences that can form a stable double‐stranded region through Watson–Crick base pairing. Owing to the unique properties of DNA, it has been used as an ideal motif to decorate the NPs for building various aggregates with a predictable and precise function,^[^ ^68^ ^]^ which would contribute to the improved resolution and sensitivity of diagnostics, subsequently effectively guiding therapy.^[^ ^69^, ^70^ ^]^ Zhang's group reported a work of in situ self‐aggregation of NIR‐II nanoprobes for improving image‐guided cancer surgery (**Figure** **7**a). The downconversion NPs (DCNPs) were modified with complementary DNA (L1 or L2) and targeting peptides (follicle‐stimulating hormone [FSH_β_]) to form DCNPs‐L1‐FSH_β_ as well as DCNPs‐L2‐FSH_β_. The initial size of DCNPs‐L1‐FSH_β_ was about 8–17 nm,; once the complementary DCNPs‐L2‐FSH_β_ was introduced, the aggregates of DCNPs were formed with the size distribution of ≈100–500 nm. After two‐stage sequential injections at an interval of 8 h, the tumor‐to‐normal tissue (T/N) ratio of aggregates was maintained at ≈12.5 within 20−28 h after the first injection, which was fivefold higher than the single injection of DCNPs‐L1‐FSHβ (T/N = 2.5 in 20 h) (Figure 7b). The long stable tumor retention of nanoprobes favored detection and resection of ovarian tumor, especially metastatic lesions, where less than 1 mm was thoroughly removed (Figure 7c). In addition, the enzyme‐triggered self‐aggregation of NPs through the DNA hybridization process was also achieved. Nie's group developed a nanoprobe by conjugating complementary DNA through the MMP‐2‐responsive peptide on the surface of AuNPs for enhanced imaging‐guided chemophotothermal therapy.^[^ ^69^ ^]^ The nanoprobes would form aggregates rapidly through DNA hybridization under the overexpressed MMP‐2, leading to the hydrodynamic diameter of the nanosystem to increase significantly from 73.2 ± 18.8 nm to 1.8 × 10^6^ ± 1.4 × 10^5^ nm. The self‐aggregation of the nanoprobes strategy can significantly enhance NIR absorption, which is conducive to deep tissue imaging and treatment.

### 3.3 Interparticle Crosslinking Reaction‐Driven Self‐Aggregation

In addition to the noncovalent interactions, the interparticle crosslinking interactions such as bio‐orthogonal click reactions with the characteristics of fast, exquisitely specific, and high‐yielding covalent reactions have also been extensively used for the NP's self‐aggregation. The bio‐orthogonal click reactions can be carried out in a biological environment without disrupting the natural biochemical process and those commonly used in organisms are Staudinger ligation, click reactions, inverse electron‐demand Diels−Alder reaction (IEDDA), and CBT‐Cys condensation reaction; they can effectively facilitate self‐aggregations of NPs.

**Figure 8:**
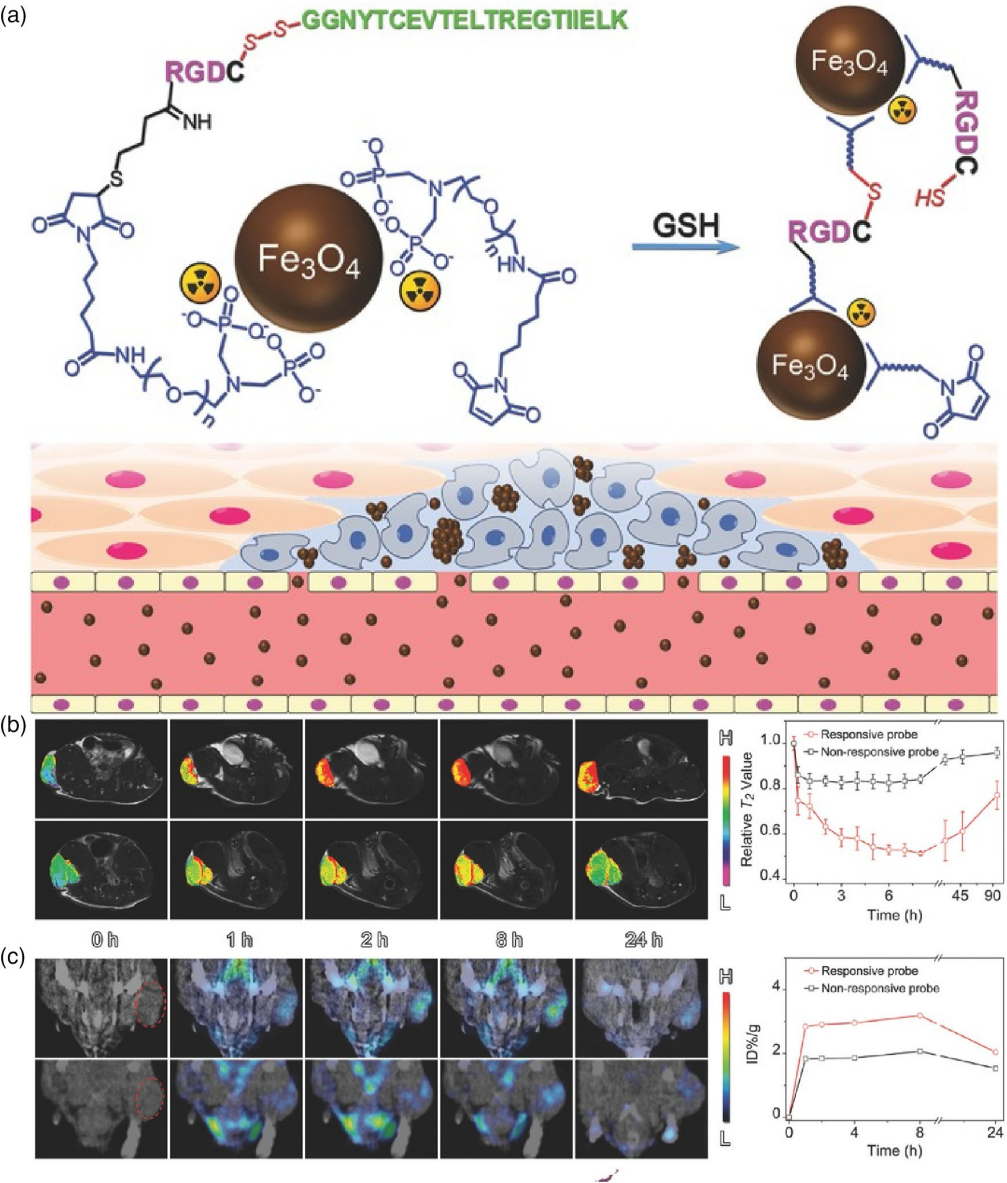
a) The illustration of GSH‐responsive nanoprobe labeled with ^99m^Tc which can in situ crosslink Fe_3_O_4_ to self‐aggregate into large‐sized IONPs through interparticle crosslinking reaction for enhanced dual‐modality imaging. b) T2‐weighted MRI and c) SPECT/CT imaging of tumor‐bearing mice after intravenous injection of the responsive probe (top) and the nonresponsive probes (bottom) at different time points, as well as their corresponding quantified values (right). Reproduced with permission.^[^ ^72^ ^]^ Copyright 2017, Wiley‐VCH.

Attributed to the biosecurity of iron oxide NPs (IONPs), it has been regarded as a promising contrast agent for tumor MRI. Moreover, the IONPs with size larger than 10 nm exhibit a stronger T2 effect due to the high saturation magnetization.^[^ ^71^ ^]^ To achieve signal enhancement, a series of stimuli−responsive NPs which can in vivo self‐aggregate for enhanced tumor MRI have been exploited.^[^ ^72^, ^73^, ^74^ ^]^ Gao and Yang's groups developed a GSH‐responsive nanoprobe labeled with ^99m^Tc for in situ crosslinking of Fe_3_O_4_ to aggregate into large‐sized IONPs for enhanced dual‐modality imaging (**Figure** **8**a). The nanoprobe was modified with a peptide sequence in which the RGD peptide and a self‐peptide as a “mark of self” were linked via a disulfide bond. The self‐peptide serves as a stealth coating for prolonging the circulation time of the NPs. After the cleavage of the self‐peptide by high concentration of GSH within the TME, the RGD moieties were exposed to targeting to overexpressed α_v_β_3_ on the surface of cancer cells. Subsequently, the thiol groups remaining on RGD moieties react with maleimide moieties from adjacent particles to crosslink the particles (initial size of 7.5 ± 0.6 nm) in situ to form large nanoaggregates with a size of 69.2 nm. Upon this process, compared with noncrosslinkable Fe_3_O_4_ nanoprobes, the self‐aggregation strategy can increase tumor MRI contrast by more than 3 times in vivo (Figure 8b) and also enhance SPECT‐imaging capabilities (Figure 8c). Therefore, the click reactions‐driven self‐aggregation strategy exhibits great potential for designing advanced probes for sensitive and dual‐modality tumor imaging.

**Figure 9:**
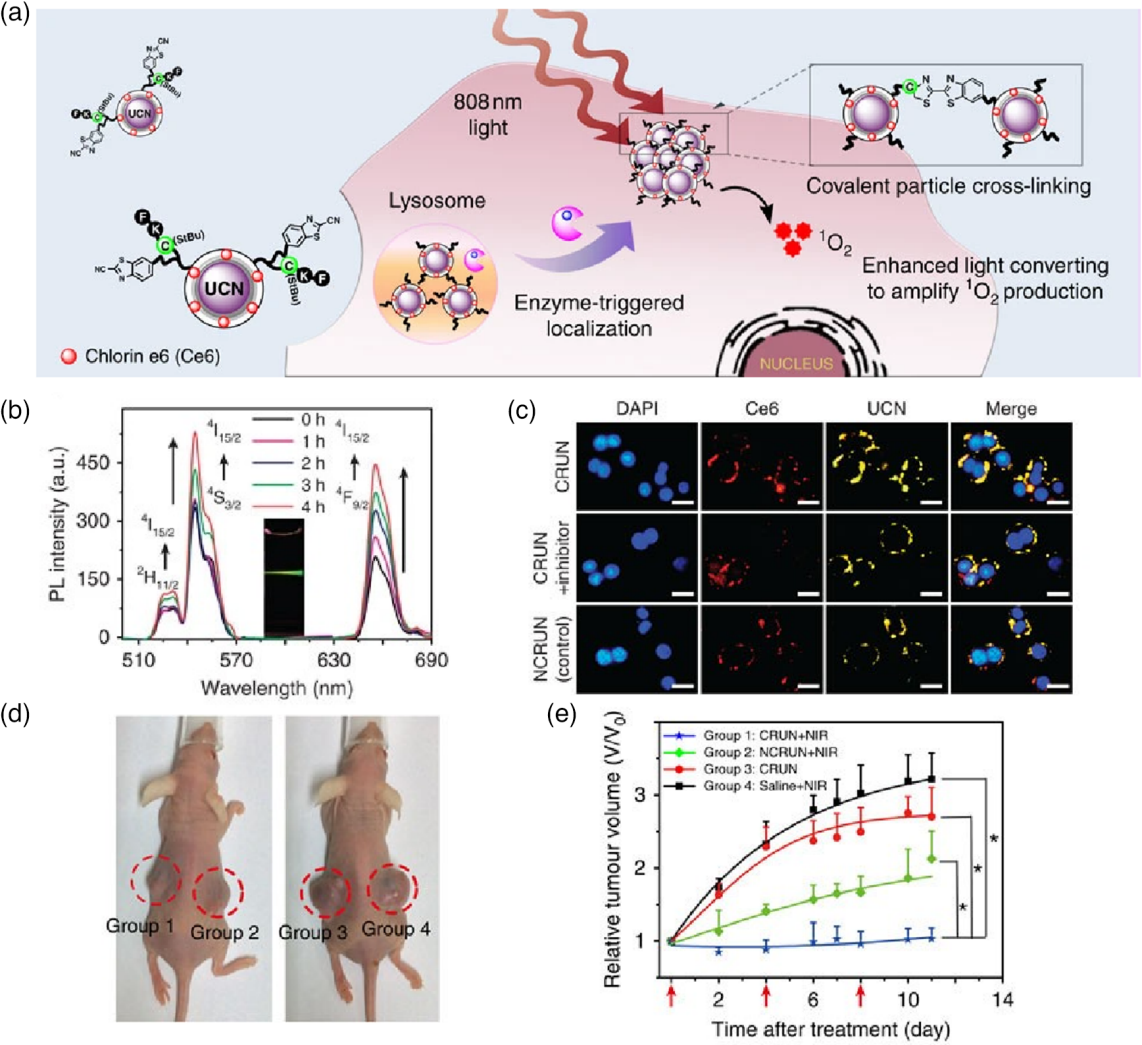
a) Scheme illustration of tumors‐specific UCNs (CRUN) modified with peptide and loaded chlorin‐e6 (Ce6), which enabled enhancing tumor‐specific accumulation and retention through in vivo CBT‐Cys condensation reaction for enhanced PDT. b) Upconversion luminescence of CRUN with cathepsin‐B treatment at different time intervals. c) Confocal imaging of different groups treated with HT‐29 cells. Scale bar, 20 mm. d) Photographs of tumor‐bearing mice after treatment with CRUN, NCRUN, and CRUN without NIR light irradiation and saline. e) The tumor growth curves of different treatment groups under 808 nm laser irradiation. Data are means ± SD (*n* = 8 mice per group). Statistical significance is assessed by a student's t‐test (heteroscedastic, two‐sided). *p < 0.05. Reproduced under the terms of the CC‐BY 4.0 license.^[^ ^77^ ^]^ Copyright 2016, The Authors, published by Springer Nature.

Over the past decades, upconversion nanocrystal (UCN) has been widely used for PDT; it is a transducer with the capability of deep tissue penetration due to its unique nonlinear photon upconverting process upon NIR light irradiation.^[^ ^75^, ^76^ ^]^ Therefore, boosting the light conversion efficiency of UCN is considered to be a reliable strategy that can effectively enhance the efficacy of PDT. Xing and coworkers reported TME‐sensitive UCNs (CRUN) modified with peptide and loaded chlorin‐e6 (Ce6), which enabled enhancing tumor‐specific accumulation and retention through the in vivo crosslinking reaction.^[^ ^77^ ^]^ The peptide was cleaved by the overexpressed cathepsin B in cancer cells to expose free Cys, resulting in its crosslinking with CBT on neighboring NPs; thus, the nanoaggregates were formed with size of about 1,580 nm (**Figure** **9**a). After incubation with cathepsin B, luminescence intensity was enhanced 2.2‐fold higher at 655 nm (Figure 9b). As shown in Figure 9c, the cathepsin B‐overexpressed HT‐29 cells treated with CRUN represented a significant luminescence intensity increase, compared with that of the noncrosslinking control group (NCRUN). Therefore, the nanoaggregates realized an enhanced upconversion emission under 808 nm laser irradiation and then further amplified the singlet oxygen (ROS) generation of Ce6 for enhanced PDT (Figure 9d,e). In addition, the CBT‐Cys condensation reaction can also be used to drive the self‐aggregation of NPs for enhancing PTT efficacy. AuNPs have largely been regarded as promising PTT therapeutic agents. However, only AuNPs with a size larger than 50 nm possess strong NIR absorption, which is necessary for PTT.^[^ ^78^ ^]^ Wang's group developed the in situ self‐aggregation of AuNP nanoplatform for enhanced PTT.^[^ ^79^ ^]^ After AuNPs that modified with RVRR peptide were internalized by cancer cells, the intracellular high concentration of GSH and overexpressed trans‐Golgi protein furin triggered CBT‐Cys condensation reaction to form aggregates with a diameter of 103.5 ± 12.3 nm. The aggregates showed increased absorption in the NIR region and significantly improve their photothermal behavior and PTT efficiency.

## 4 Challenges and Outlook

In this review, the driving forces for in situ size increasing of NPs in living cells and animals for biomedical applications have been summarized. Some molecules or relatively small‐sized NPs can penetrate into the tumor site and grow into a specific large‐size superstructure upon the TME stimuli, which shows the advantages of high retention rate and desirable biodistribution in tumor lesion for high‐performance imaging and effective tumor treatment.

In addition to cancer theranostics, the in situ size increasing of NPs is also applied in the detection of other diseases.^[^ ^80^ ^]^ Wang's group developed a novel chlorophyll–peptide conjugate which can be cleaved by enzymatic in vivo to form ordered J‐type assembled aggregates for specific and sensitive imaging of bacterial infection.^[^ ^81^, ^82^ ^]^ By conjugating the pH‐sensitive zwitterionic imidazole groups onto the ultrasmall luminescent GNPs (PMIZ‐AuNPs), Liu's groups constructed an effective ultrasound contrast with pH‐induced charge reversal and in situ aggregation properties to diagnose acidosis‐induced early kidney injury.^[^ ^83^ ^]^ However, in other diseases, such as atherosclerosis, neurodegenerative diseases, diabetes, and aging, the in situ size increase strategy is less involved and further development and research are necessary. Although great progress has been made in the size‐increasing system, there are still some crucial issues and challenges to be further optimized and solved.

First, the rational design of multifunctional molecules or relatively small‐sized NPs to form robust superstructures in vivo is still challenging. The key factor of the precursor design is the pathological abnormality of the disease site; under the stimuli of abnormality, the size of NPs tends to increase. However, imputed to the heterogeneity of tumor sites, the level and activity of stimuli may differ across types and species. Therefore, it is necessary for an in‐depth study and understand the pathological properties of the disease site at the molecular level for further exploiting new ways for size increasing in vivo. In addition, the few chemical reactions that can be used to trigger size increasing based on abnormalities in the tumor lesion site limit the development of the design method.

Second, the expected functions of well‐designed precursors may be attenuated by the complex physiological environment in vivo. The concentration of the precursors in the tumor site is restricted to its compact extracellular matrix (ECM) and elevated interstitial pressure. The dense ECM will prevent the precursors’ penetrating depth into the tumor to some extent. When entering tumor sites, due to high interstitial pressure, they will be easily pumped out, resulting in a shortened residence time at the tumor site. Therefore, it is of great importance for improving the sensitivity and response time of size increasing process. Using the bio‐orthogonal reactions to improve the response time can be an ideal strategy.

Third, although some exciting progress has been made in size‐increasing strategy, the size increasing of NPs in vivo lacks effective direct evidence. The current strategy is to use bio‐TEM to verify the size increasing of NPs in pretreated cells or tissues,^[^ ^84^ ^]^ but when it comes to the self‐assembly of small molecules, which might be misled by inherent nanostructures of cells (or tissues), it is urgently needed to exploit high‐resolution tools and strategies to illustrate the dynamic behavior.^[^ ^85^, ^86^ ^]^ What is more, the deepening understanding of the dynamic behavior of size increasing in vivo will also contribute to the rational design of precursors with a theoretical basis. In addition, the surface effect and morphology of NPs will change accordingly during the size‐increasing process, and the mechanism and comprehensive quantitative relationship between the large‐sized structure and cancer cell death should be further explored. Furthermore, whether the size increase can cause tumor immune response and even enhance its antitumor immune efficacy should be further identified.

Finally, improving the targeting of NPs and reducing its potential toxicity caused by nonselective accumulation in normal organs are other important aspects. Biomimetic NPs have attracted considerable attention with the unique advantages of better biocompatibility, low immunogenicity, and tumor‐targeting capacity for tumor theranostics. Therefore, constructing biomimetic NPs based on native cells or exosomes for in situ size increasing to improve the targeting ability can be a promising strategy. In addition, the in situ size increasing strategy will significantly increase the stability and retention time of nanoagents (contrast agents or therapeutic agents) in the disease site, but whether it can be metabolized quickly and the risk of long‐term toxicity should be considered. Therefore, designing nanoagents which can achieve size increasing to facilitate their function in the tumor site and then deforming into small particles for fast elimination may be a potential solution.^[^ ^87^ ^]^ The evaluation of pharmacokinetics and biosecurity in biological systems is urgently needed, which will greatly help in clinical translation toward diagnostics and therapeutics of diseases.

## Conflict of Interest

The authors declare no conflict of interest.
